# Pathogens associated with acute diarrhea, and comorbidity with malaria among children under five years old in rural Burkina Faso

**DOI:** 10.11604/pamj.2021.38.259.15864

**Published:** 2021-03-12

**Authors:** Palpouguini Lompo, Marc Christian Tahita, Hermann Sorgho, William Kaboré, Adama Kazienga, Ashmed Cheick Bachirou Nana, Hamtandi Magloire Natama, Isidore Juste Ouindgueta Bonkoungou, Nicolas Barro, Halidou Tinto

**Affiliations:** 1 Institut de Recherche en Science de la Santé, Unité de Recherche Clinique de Nanoro, Ouagadougou, Burkina Faso,; 2 University of Ouagadougou I, Prof. Joseph Ki Zerbo, Ouagadougou, Burkina Faso,; 3 Laboratoire National de Santé Publique, Ouagadougou, Burkina Faso

**Keywords:** Diarrhea, infectious, pathogens, bacteria, rotavirus, parasite, malaria, Burkina Faso

## Abstract

**Introduction:**

acute diarrhea in children under five years is a public health problem in developing countries and particularly in malaria-endemic areas where both diseases co-exist. The present study examined the etiology of childhood diarrhea and its comorbidity with malaria in a rural area of Burkina Faso.

**Methods:**

conventional culture techniques, direct stools examination, and viruses´ detection by rapid tests were performed on the fresh stools and microscopy was used to diagnose malaria. Some risk factors were also assessed.

**Results:**

on a total of 191 samples collected, at least one pathogen was identified in 89 cases (46.6%). The proportions of pathogens found on the 89 positive stool samples were parasites 51.69% (46 cases), viruses 39.33% (35 cases), and bacteria 14.61% (13 cases), respectively. The relationship between malaria and infectious diarrhea was significant in viral and parasites causes (p=0.005 and 0.043 respectively). Fever, vomiting and abdominal pain were the major symptoms associated with diarrhea, with 71.51%, 31.72% and 23.66% respectively. The highest viral diarrhea prevalence was reported during the dry season (OR=5.29, 95% CI: 1.74 - 16.07, p=0.001) while parasite diarrhea was more encountered during the rainy season (OR=0.41, 95% CI: 0.33 - 0.87, p=0.011).

**Conclusion:**

Giardia spp and rotavirus were the leading cause of acute diarrhea in Nanoro, Burkina Faso with a predominance of rotavirus in children less than 2 years. Parasite and viral diarrhea were the most pathogens associated with malaria. However, the high rate of negative stool samples suggests the need to determine other enteric microorganisms.

## Introduction

Diarrheal disease remains a worldwide public health problem and represents the second leading cause of death in children under five years old. Indeed, diarrhea kills around 760 000 children under five yearly worldwide, with the majority of cases in sub-Saharan Africa [1, 2]. Studies conducted around the world have identified enteric bacteria, viruses and parasite as the main infectious causes of diarrheal illness [3-6]. In Africa, apart from protozoan and bacteria, it has been demonstrated that rotaviruses play a leading role in the occurrence of diarrheal illness in children [7, 8]. In malaria endemic settings, most children under five with malaria present with diarrhea. A review of malaria morbidity surveys based on hospital data demonstrated an association between malaria and diarrhea in 6%-40% of clinical malaria cases [9, 10].

In Burkina Faso, few studies have been conducted and consistent data are still scarce. In addition, most of the available data are from studies carried out in urban or semi-urban areas, therefore the results cannot be applied in rural areas [8, 11-13]. Poor hygienic conditions, lack of drinking water combined with poverty and the high illiteracy rate in rural areas can influence the profile of infectious diarrhea [1, 3]. Finally, the magnitude of the comorbidity diarrhea-malaria is not well known in the country although the overlap of their respective endemicities suggests their co-occurrence. The present study aims to identify the infectious etiologies and prevalence of childhood diarrhea, and assess their interaction with malaria in areas of Nanoro, Burkina Faso.

## Methods

**Study setting, design and procedures:** a cross-sectional descriptive study was conducted from March 2012 to March 2014 in the rural health district of Nanoro to investigate the etiologies of diarrhea in children aged below five years old, and their coinfection with malaria. The study was carried out in two health centers located in the Nanoro Health and Demographic Surveillance System (HDSS) catchment area. The Nanoro health district had around 150,000 inhabitants with 22.9% of children under five years living in an area of highly seasonal malaria transmission which overlaps with the rainy season [14, 15]. Diarrheal diseases generally occur during the cold and dry season (December - February). Since October 2013, Burkina Faso has introduced pentavalent rotavirus vaccine (RotaTeq) in the national immunization program in order to reduce the morbid-mortality due to rotavirus infection [16]. All children attending the two health centers and fulfilling the inclusion criteria (children under 5 years suffering from diarrhea) were asked to participate in the study. After obtaining a written informed consent, a questionnaire was used to record information on medical history; vaccination status for rotavirus vaccine and antimalarial/antibiotics treatment and physical examination was performed. Blood sample was taken by finger prick for malaria diagnosis while fresh stool specimens were collected using sterile flaks for the pathogens identification.

**Laboratory procedures:** all samples collected were proceeded within 1 hour. Thick and thin blood films were Giemsa stained and examined for detection of Plasmodium species according to standard procedures [17]. The results were expressed as asexual parasites per microliter using the World Health Organization (WHO) estimated number of white blood cell (8000/µl). A double reading system was used and in case of discrepancies a third reader was used. A direct examination of fresh stool was done in order to detect protozoa, eggs and cysts. Rotavirus and adenovirus were detected by using immuno-chromatographic assay (SD Bioline Rota/Adeno; Standard Diagnostics, Inc. Kyonggi-Do, South Korea). Stools were subsequently cultured on appropriate media (Eosine Methylen Blue and Hektoen agar, bioMérieux, Marcy-l´Etoile, France) and incubated at 35-37°C for 24 hours in aerobic conditions. The disc diffusion method was done for antimicrobial susceptibility testing according to Clinical and Laboratory Standard Institute (CLSI) guidelines [18]. *Shigella spp*. and *Salmonella spp*. isolates were serotyped using specific antisera from BioRad, France, following the Kauffmann-White scheme [19]. All the isolates and fresh stools were stored at -80°C for further investigations.

**Statistical methods and definitions:** data were double entered, validated and cleaned using Epidata version 3.1. Statistical analysis was performed using STATA Version 10 (STATA Corporation, College Station, TX, USA). Diarrhea was categorized as follow: acute diarrhea (lasting less than 14 days), dysentery (presence of blood or mucus), and persistent diarrhea (more than 14 days) [20]. Fever was defined as axillary temperature ≥37.5°C while malaria was defined as the presence of asexual Plasmodium species parasites confirmed by microscopy plus fever or history of fever in the last 48 hours. Infectious diarrhea was defined when at least one enteric pathogen (parasite, bacteria or virus) was identified in the stool. Descriptive statistics were performed using proportions for categorical variables. Categorical variables univariate analyses were appropriately assessed for significance using the chi-square test or Fisher exact test.

## Results

A total of 191 children were enrolled. Demographic data, clinical aspect and laboratory results of study population is summarized on Table 1. The overall rate of infectious diarrhea was 46.6% (89/191). About half of the pathogen encountered was parasites followed by the viruses (40%). The bacteria were responsible for diarrhea in less than 15% of the cases. The most frequent parasites were *Giardia sp*with 45.65% of cases in mono-infection, followed by *Entamoeba coli* and *Hymenolepis nana*. Among the viruses detected, rotavirus represented one-third of the infections while the proportion of adenovirus was less than 10%. The most frequent isolated bacteria were *Shigella spp*. and the enteropathogenic *Escherichia coli* (EPEC) in 8% and 6% respectively. Fever (71.51%) was the first reason of health facility attendance followed by vomiting (31.7%) and abdominal pain (23.7%) (Table 1).

**Table 1:** demographic data, clinical aspects and summary of laboratory results

Parameter	Number	Percentage (%)
**Age groups**		
Children < 24 months	131	68.59
Children ≥ 24	55	28.79
Not specified	5	2.62
Mean age in months (SD)	19.02	11.74
**Gender**		
Male	110	57.59
Female	81	42.41
**Symptoms**		
Fever	133	71.51
Vomiting	59	31.72
Abdominal pain	44	23.66
Dehydration	5	2.69
Edema	1	0.54
**Microbes identified**		
Parasites	46	24.08
Viruses	35	18.32
Bacteria	13	6.81
**Blood smears for malaria diagnosis**		
Positive	60	31.41
Negative	131	68.59
Parasites density ≤ 400/μl	7	11.67
Parasites density > 400/μl	53	88.33

The mean age of children was 19.02±11.74; Parasites and viruses were more common pathogens isolated in stool samples; Frequency of comorbidity diarrhea and malaria was encountered in 31.41%; More than 50% presented high Parasites density

The search of association between diarrhea and the described factors showed that very younger age (< 2 years) was strongly associated with viral diarrhea (OR = 7.22, 95% CI: 1.64 - 31.73, P < 0.001) while children above 2 years old were more infected with parasites than virus (OR = 0.21, 95% CI: 0.10 - 0.44, P < 0.001). Bacterial infection was not associated with patient age (Table 2). Malaria in co-infection with diarrhea is shown in Table 3. All cases of malaria were due to *Plasmodium falciparum*. The relationship between malaria and infectious diarrhea was statistically significant in viral and parasites causes (P = 0.005 and 0.043 respectively). A seasonal pattern of the distribution of associated pathogens was seen with parasite infection which was more associated with rainy season and the virus infection which was associated with the cold and dry season, (OR = 5.29, 95% CI: 1.74 - 16.07, P = 0.001 and OR = 0.41, 95% CI: 0.33 - 0.87, P = 0.011 respectively) (Table 4). In opposite, this such association was not observed with the bacterial diarrhea. The majority of the bacteria isolated were susceptible to ciprofloxacin, gentamycin, and ceftriaxone but were resistant to ampicillin and trimethoprim + sulfamethoxazole.

**Table 2:** association between age group and microbes

Pathogens	Age group (months)		Odds Ratio	p-value
	< 24	≥ 24	OR * (95%CI)	
Viruses	32 (24.43)	2 (3.64)	8.56 (1.97 - 31.14)	0.004
Bacteria	10 (7.63)	3 (5.45)	1.43 (0.37 - 5.41)	0.596
Parasites	19 (14.50)	25 (45.45)	0.20 (0.09 - 0.41)	<0.0001

* Children with age ≥ 24 were reference category

**Table 3:** interaction between infectious diarrhea and malaria

Diarrhea causes	N (%)	% with malaria	p-value
**Viral diarrhea**			
Yes	35 (18.32)	11.43	0.005
No	156 (81.68)	35.90	
**Bacterial diarrhea**			
Yes	13 (6.81)	23.08	0.502
No	178 (93.19)	32.02	
**Parasite diarrhea**			
Yes	46 (24.08)	43.48	0.043
No	145 (75.92)	27.59	
**Diarrhea with unknown causes**			
Yes	104 (54.45)	33.65	0.466
No	87 (45.55)	28.74	

Significant association between parasitic and viral diarrhea with malaria

**Table 4:** influence of season on the occurrence of pathogens

Type of etiology	Dry season	Rainy season	OR * (95% CI)	p-value
Virus	31 (27.68)	4 (5.06)	7.17 (2.41 - 21.29)	<0.0001
Bacteria	9 (8.04)	4 (5.06)	1.63 (0.48 - 5.52)	0.426
Parasites	19 (16.96)	27 (34.18)	0.39 (0.19 - 0.77)	0.007

* Rainy was the reference category

## Discussion

The objective of the present study was to determine the prevalence of infectious acute diarrhea and its associated pathogens among children under five years old in rural Burkina Faso. The results showed that parasitic infection was present in about half of the acute diarrhea cases. This finding contrasts with results from studies conducted in urban areas of the country, where viruses and bacteria were more prevalent [7, 8]. If the high prevalence of *Giardia sp*among our study samples supports the hypothesis of general poor hygienic conditions in rural areas [21, 22], more detailed investigations, which include environmental factors - in addition to others diseases, should help us to improve understanding of the local ecology of diarrheal disease. Contrary to parasitic infections, the prevalence of viral acute diarrhea in our study was low compared to reports of other studies conducted in Burkina Faso and elsewhere [3, 4, 13, 23, 24]. Indeed, a study conducted in Ouagadougou, Burkina Faso and other African countries in 2013 reported a higher prevalence of viral infections [3, 6-8]. This difference could be explained by the higher proportion (83%) of children under two years old enrolled in these studies. Although these studies were different in their design, they showed that rotavirus remains an important cause of infectious diarrhea mostly in children under two years old with some variability in the prevalence that could be related to both the number of children in this age group enrolled and the period covered by the study [3, 6, 7, 13, 23].

Finally, very few bacteria species were isolated in our study regardless of the age of the patients. This finding was surprising because in previous studies of childhood diarrhea, bacterial causes were the most important after viral causes [3, 6, 23]. Self-medication with antibiotics is very common in many parts of Africa including Burkina Faso, and this could be the main cause of such low detection of bacteria in the stools [5, 7, 8]. Indeed, a high proportion of the children in this study had a history of antibiotic use prior to stool sampling, which was already reported in a study conducted in Tanzania [7]. It is also important to mention that not all known diarrheagenic bacteria were included in our panel of exploration. Only *Salmonella spp, Shigella spp*. and EPEC were investigated. Out of the thirteen cases of bacterial diarrhea reported in our study, *Shigella spp*. was predominant. The role of *Shigella spp*. in childhood diarrhea deserves further attention [25, 26]. Furthermore, the resistance of these bacteria to some antibiotics such as cotrimoxazole and nalidixic acid may worsen the situation and this needs to be explored carefully. A similar conclusion was reported in the study conducted in Ouagadougou regarding the resistance of Shigella to cotrimoxazole, which is the first line drug recommended for the treatment of childhood diarrhea in many countries including Burkina Faso [7, 8].

Fever along with abdominal pain and dehydration were the common symptoms reported by our study participants. Similar findings were already observed in a study conducted in Ouagadougou in 2010 and elsewhere [12]. In other words, fever is more frequent in childhood diarrhea especially in the study setting where malaria is endemic. Indeed, one third of children suffering from diarrhea were also diagnosed with malaria. Nevertheless, diarrhea and malaria co-infection was more frequent in viral and parasitic cases. Studies in Guinea-Bissau and Cameroon have previously mentioned the association between malaria parasitemia and childhood diarrhea but their statistical association was not assessed [9, 10]. However, a study in Ghana found an association between diarrhea and clinical malaria but this was not specific to a detected enteropathogenic organism [3]. This is an indication that acute diarrhea management (diagnosis and treatment) should not exclude the diagnosis of malaria.

In our study, the occurrence of pathogens associated with diarrhea was significantly influenced by the season. Hence, viral diarrheas were more frequent in the dry and cold season, from November to February, while parasitic infections were more frequent during the rainy season, from June to October. Similar findings were highlighted by Bonkoungou et al for viral diarrhea, particularly with rotavirus in a study conducted in Ouagadougou [12]. In Tanzania a study reported a frequency of rotavirus throughout the year, but with two peaks in February and December, probably due to the difference in climate [26].

## Conclusion

Our study gives an overview of pathogens associated with childhood diarrhea in Nanoro, a rural setting of Burkina Faso. Thus, parasites and viruses were the more frequently incriminated pathogens with rotavirus and *Giardia spp*. respectively. These two pathogens are also the most associated with malaria. The bacterial isolates were rare. The main symptoms are also frequent with malaria infection making the biological diagnostic a crucial tool to be used, as well as a refining of well guideline for Integrated Management of Childhood Illness. Further investigations including the genotyping of virus strains encountered and the detection of a large spectrum of microorganisms are needed.

### What is known about this topic

- The prevalence of diarrhea in children mostly investigated in the urban areas in Burkina Faso;
- The common pathogens incriminated in diarrheal diseases.

### What this study adds

- The infectious etiologies and their prevalence in childhood diarrhea in rural areas in Burkina Faso were different to the urban areas;
- The significance of the interaction between diarrheal pathogens and malaria in Burkina Faso, a malaria endemic area was seen in viral and parasite pathogens;
- The main etiologies were associated with season, mostly the parasites like Giardia spp in the rainy season.
